# On the Term Set’s Semantics for Pairwise Comparisons in Fuzzy Linguistic Preference Models

**DOI:** 10.3390/e25050722

**Published:** 2023-04-26

**Authors:** Ana Nieto-Morote, Francisco Ruz-Vila

**Affiliations:** 1 Project Engineering Department, Polytechnic University of Cartagena, c/Dr. Fleming, s/n, 30202 Cartagena, Spain; ana.nieto@upct.es; 2 Department of Electric Engineering, Polytechnic University of Cartagena, c/Dr. Fleming, s/n, 30202 Cartagena, Spain

**Keywords:** multicriteria decision making, pair-wise comparisons, fuzzy preference relations, semantic rules, referential term, linguistic modifiers

## Abstract

The main objective of this paper is the definition of a membership function assignment procedure based on inherent features of linguistic terms to determine their semantics when they are used for preference modelling. For this purpose, we consider what linguists say about concepts such as language complementarity, the influence of context, or the effects of the use of hedges (modifiers) on adverbs meaning. As a result, specificity, entropy and position in the universe of discourse of the functions assigned to each linguistic term are mainly determined by the intrinsic meaning of the hedges concerned. We uphold that the meaning of weakening hedges is linguistically non-inclusive because their semantics are subordinated to the proximity to the indifference meaning, whereas reinforcement hedges are linguistically inclusive. Consequently, the membership function assignment rules are different: fuzzy relational calculus and the horizon shifting model derived from the Alternative Set Theory are used to handle weakening and reinforcement hedges, respectively. The proposed elicitation method provides for the term set semantics, non-uniform distributions of non-symmetrical triangular fuzzy numbers, depending on the number of terms used and the character of the hedges involved. (This article belongs to the section “Information Theory, Probability and Statistics”).

## 1. Introduction

In the process of group decision-making under a linguistic environment, each expert generally needs to compare a set of alternatives and/or criteria and usually constructs a linguistic preference relation using some linguistic terms [1]. Since the work of Labov [2] and Lakoff [3] questioned the assumption that meanings are precise, it has been accepted that they have a certain degree of vagueness, such that the boundary of the application of a linguistic term is a region where it gradually moves from being applicable to non-applicable. Developments in fuzzy set theory offer a formal treatment of the vagueness of natural language concepts, and its usefulness has been tested in many applications.

There are two classical ways to address this issue with a fuzzy linguistic approach [4]:

- The use of symbolic models, which implies that a membership function is not necessarily numerical but that a qualitative label is sufficient to get an ordered set; the order structure is used to implement symbolic calculations;
- The use of membership functions-based models, which implies operating directly with them through the Extension Principle and interval arithmetic. Usually, they use context-free grammar to generate the linguistic labels required to define the linguistic variables.

For the first, semantics depends only on order, which entails a loss of information when, after computing processes, results should be linguistically interpreted. To avoid this drawback, a two-tuple linguistic model [5] was developed, and during the last two decades, it has been widely improved and used. Nevertheless, they are still considered inflexible models [6] that cannot handle language complementarity, and several research lines are being developed to enhance the adaptation to this circumstance, for example, to make suitable the use of their computation procedures with the use of context-free grammars [7] or to consider that different people have different semantics understandings, taking into account the psychophysical state of the human being [8,9]. Even new models which combine probability theory and fuzzy set theory have been designed [10].

For the second, membership grades are supposed to be numerical, and the main drawback stems from the fact that the use of interval arithmetic increases the vagueness of the outcomes, making them hard to interpret. To improve the performance of these models, Lodwick and Jenkins introduced the concept of constrained arithmetic [11], which has been profusely covered in the literature [12,13], and some important related issues, such as additive consistency definition, have been analyzed and adapted [14] to avoid the unintended excessive increase of vagueness.

Following the latter line of work, our contribution focuses on the semantics of membership functions, whose incorrect definition is also a source of undue increase in the vagueness of processing results in computing with words procedures. Moreover, it shows a way to obtain different meanings for the same linguistic term in different contexts, which is what language complementarity means.

When membership functions-based models are used, there should be some operational definition of these numbers. For this task, Zadeh [15] suggested two ways to define a membership function: by enumerating and assigning membership values in a finite domain or by being a continuous and differentiable function of a numerical value representing a measurable property (the variable of the function). However, the real problem remains even when the conditions described above are satisfied: where do the assigned values or the function parameters come from? If fuzziness is not well-interpreted, the assigned values mentioned above seem to be arbitrary and artificial. In such a case, in what sense are the system’s results, which are determined by these initial assignments, better than random choices? We assume that the application of the linguistic approach to the development of decision-making models should consider the inherent semantics of every linguistic term as the keystone of system formalization.

If we think that fuzzy logic is a valid tool to handle the inherent fuzziness that human reasoning has, we need a way to explain what “fuzziness” is within a given context in order to assign different extensions to a concept intention. This problem is related to linguistic complementarity [16], which states that it is not possible to unequivocally link the description and the interpretation of a linguistic term due to the fact that meanings require a concrete expression, but their interpretation might evolve and be non-singular. In the same way that linguistics uses lexicographical tools as dictionaries to determine how to interpret the meaning of a term in certain contexts, the achievement of such an objective when a linguistic approach is used in decision-making support systems implies the definition of a procedure to correlate a linguistic term with a membership function, in a given semantic space.

Thus, our objective is to formally relate the concerned concepts within a given context with the functions used to quantify the corresponding degree of membership in a psychologically acceptable viewpoint [17], although we know that there could be not only one plausible solution because we are working on a scenario where subjectivity is a key issue.

For preference relations and pairwise comparisons, sentences with the pattern “*C_i_ is ‹adverb› ‹adjective› than C_j_*” are commonly used. Mathematically, each *‹adverb› ‹adjective›* pair is a linguistic value quantifying the linguistic variable that defines the object of the comparison. For example, the linguistic variable “*Relative Importance*” could be quantified by terms such as “*Strongly More Important*” or “*Less Important*”.

In most cases, for preference modelling, the complete linguistic term set is elicited through symmetrical triangular membership functions uniformly distributed in the universe of discourse [18,19,20,21]. Two main reasons are usually suggested for this wide-spread use: firstly, the simplest form that one can think of for modeling the graduality is the linear transition between the support and the core of the membership function; secondly, the problems dealing with vague predicates are less concerned with precision, and they are more of a qualitative type and are thus generally written as linearly as possible. We are aligned with Pedrycz’s [22] considerations in the sense that the simplicity does not justify, by itself, the “almost” universal use of this type of function, and we also think that the shape of the membership functions is not only a matter of precision, even in the field of approximate reasoning. Consequently, it is necessary to base the use of triangular membership functions (or any other shape) within an adequate theoretical frame.

Concerning the uniformity of the distribution, the assignment of a membership function based on the granularity of the term set and the position of each label in an ordered structure [23] is not sufficient to obtain a psychologically acceptable elicitation. We are aligned with Giles’s [24] point of view that defends a different approach called the semantic/pragmatic approach, where semantics must be understood as the abstract meaning and pragmatics as the concrete meaning that an agent gives to a linguistic term.

The aim is to start with an analysis of the immediate practical meanings of the concepts involved and, by explicating these meanings, to deduce the properties of the concepts.

It is generally accepted that the set of linguistic terms or labels has at least two levels:

- The primary term set provides the comparative forms of the adjective (e.g., “*More Important*”, “*Equally Important*”, and “*Less Important*”;
- The terms obtained by application of linguistic modifiers to the primary ones (e.g., “*Moderately More Important*”, “*Much More Important*”, or “*Extremely More Important*”).

For primary terms, Zadeh [25] said that membership functions are subjective and context-dependent, and consequently, there is no general method to determine them. We consider it essential to base all the assignment procedures on a referential term with a clear semantic meaning. For the representation of preferences in decision-support systems, we start from the elicitation of the linguistic label expressing indifference (“*Equally Important*”). The possibility theory [26] provides the abovementioned theoretical frame in the membership function selection for this referential term. The complementary operator gives the meaning of a couple of primary terms, indicating a positive or negative preference for “*More Important*” and “*Less Important*”.

In the second level, modifiers or hedges can be considered pure operators that adjust the original membership function parameters of the primary term set [27,28], or they could be regarded as inherent meaning holders. We assume this second semantic approach, combining the concept of horizon shifting [29] to elicit the reinforcement modifiers and fuzzy relational calculus [30] to introduce the weakening ones.

We resume the proposed procedure in three steps:

(a) Elicitation of the primary term set for a scenario with the maximum level of indeterminacy;
(b) Reinforcement hedges mainstreaming. First adjustment of the primary term set meaning and initial elicitation of linguistic labels, including this type of modifier;
(c) Weakening hedges mainstreaming and definitive meaning adjustments for the membership functions obtained in the previous step.

The paper is organized as follows:

- In Section 2, we define the conceptual frame where the elicitation procedure is going to be applied: the fuzzy linguistic model that we have chosen to work with preference relations and some important issues about what linguists understand by hedges or modifiers and their intrinsic characteristics;
- In Section 3, we describe the elicitation procedure: the semantic rules that give, as a result, the membership functions for each label of the term set;
- In Section 4, we confirm that the results obtained with the proposed model fit well with linguistic definitions of non-linear adjectives, weakening and reinforcement modifiers of adverbs, and language complementarity;
- Finally, in Section 5, conclusions of the contribution are remarked on.

## 2. Fuzzy Linguistic Model for Preference Relations

From a formal point of view, a linguistic variable is defined by a five-tuple {*L, S, U, G, M*}:

- *L* is the name of the linguistic variable; for example, in our case *L* = “*Relative Importance between criteria*”;
- *S* is the term set with the collection of all possible values of *L* (labels); for example, *S* = {*Less Important, Equally Important, More Important*};
- *G* is a grammar that includes the syntactic rules that generate the terms in *S*. The syntactic representation of the linguistic variable *L* is given by a set of labels associated with the term set;
- *U* is the universe of discourse where possible values of *L* are included; in our case, *U* = [0,1] × [0,1];
- *M* is a semantic rule that associates the meaning *M*(*s*) to each label *s*. The semantic characterization of the linguistic variable *L* is provided by a set of membership functions. Each membership function is associated with one label:

(1) $$M\left(s\right)=\left\{\langle u,\mu_{s}\left(u\right)\rangle ,\;|\;u\in U\right\}$$

Therefore, a linguistic variable can be seen as a fuzzy relation *L* from a set of terms *S* to a universe of discourse *U*, which assigns to pair (*s*, *u*), an element of *S* × *U* and a grade of membership.

For one label *s*, the membership function determines a fuzzy subset *M*(*s*) of *U* whose membership function is:

(2) $$\mu_{M\left(s\right)}=\mu_{L}\left(s,u\right),\;u\in U,\;s\in S\;$$

We assume the fuzzy subset *M*(*s*) of *U* as the meaning of *s* and the term *s* as the label of *M*(*s*).

From a pragmatic point of view, a linguistic variable is associated with two sets of rules that we must define:

- The syntactic rules, or the manner in which linguistic values or labels are generated;
- The semantic rules, or the computing procedure, to obtain the meaning of each label.

Dealing with the syntactic rules, when a linguistic approach is used in decision-making, experts’ opinions are expressed by choosing, among some linguistic labels or terms, those that better fit their way of thinking. Then, the selection of the term set plays an important role in the model definition.

If we consider Miller’s assessment of the number of items that human beings can handle in working memory [31], it matches with the typical granularity values used to define the linguistic term set, with no more than 13 labels. In our opinion, it means that every expert can manage this number of terms, but it doesn’t imply they use the same labels to get the same precision in language. Consequently, syntactic rules can generate more than 13 linguistic values or labels.

In the current work, we use the context-free grammar approach that defines the linguistic term set by means of a context-free grammar, *G*. A grammar *G* is a four-tuple {*V_N_*,*V_T_*,*I*,*P*}:

- *V_N_* is the set of non-terminal symbols (a non-terminal symbol is a symbol that can be reduced further by the production rules until it is reduced to a terminal symbol);
- *V_T_* is the set of terminals symbols (a terminal symbol is one that cannot be broken down further) that contains the primary terms (e.g., *low, medium, high*), the hedges (e.g., *not*, *much, very*), the relations (e.g., *lower than*, *higher than*), the conjunctions (e.g., *and*, *but*), and the disjunctions (e.g., *or*);
- *I* is the starting symbol, which is a special non-terminal symbol that appears in the initial string generated by the grammar;
- *P* represents the production rules.

The elements of the grammar, in our case, are defined in the extended Backus–Naur form as follows:

$$\begin{array}{c} V_{N}=\left\{\langle \mathrm{indifference}\rangle ,\langle \mathrm{preference}\rangle ,\langle \mathrm{equality}\rangle ,\langle \mathrm{inequality}\rangle ,\langle \mathrm{adjective}\rangle ,\langle \mathrm{relation}\rangle ,\langle \mathrm{hedges}\rangle ,\langle \mathrm{reinforcement}\rangle ,\langle \mathrm{weakening}\rangle \right\}\\ V_{T}=\left\{\mathrm{less},\;\mathrm{equally},\;\mathrm{much},\;\mathrm{more},\;\mathrm{strongly},\;\mathrm{extremely},\;\mathrm{moderately},\;\mathrm{weakly},\;\mathrm{hardly},\;\mathrm{than},\;\mathrm{important}\right\}\\ P=\{I::=\langle \mathrm{indifference}\rangle |\langle \mathrm{preference}\rangle \\ \langle \mathrm{adjective}\rangle ::=\mathrm{important}\\ \langle \mathrm{equality}\rangle ::=\mathrm{equally}\\ \langle \mathrm{inequality}\rangle ::=\mathrm{less}\;|\;\mathrm{more}\\ \langle \mathrm{hedges}\rangle =::\langle \mathrm{weakening}\rangle |\langle \mathrm{reinforcement}\rangle \\ \langle \mathrm{reinforcement}\rangle ::=\mathrm{much}\left|\mathrm{strongly}\right|\mathrm{extremely}\\ \langle \mathrm{weakening}\rangle ::=\mathrm{hardly}\left|\mathrm{moderately}\right|\mathrm{weakly}\\ \langle \mathrm{relation}\rangle ::=\mathrm{than}\\ \langle \mathrm{indifference}\rangle ::=\langle \mathrm{equality}\rangle \langle \mathrm{adjective}\rangle \langle \mathrm{relation}\rangle \\ \langle \mathrm{preference}\rangle ::=\langle \mathrm{inequality}\rangle \langle \mathrm{adjective}\rangle \langle \mathrm{relation}\rangle |\langle \mathrm{hedges}\rangle \langle \mathrm{inequality}\rangle \langle \mathrm{adjective}\rangle \langle \mathrm{relation}\rangle \} \end{array}$$

In the case of preference relationships, hedges or modifiers are the terminal symbols that determine the number of linguistic labels obtained with the grammar; we have chosen these symbols to avoid any indistinguishability (i.e., *weakly/fairly/slightly* or *very much/greatly/strongly*) and we have selected them by consensus to be considered as representative of the meaning involved. This grammar allows us to obtain a super-set of 15 linguistic labels. In any case, an expert could choose among these terms to complete the required pair-wise comparisons; the set of the different linguistic terms used by each expert is the so-called linguistic terms set *S*.

Semantics depends on context, which, in turn, includes external and internal features. External features are independent of experts’ attitudes: for example, the nature of the problem and the semantic space where the dynamics of meaning keep place [32] and its desirable properties.

In the fuzzy linguistic approach, the semantic space corresponds to an area in space where the meanings of the linguistic values or labels are expressed. Dealing with preference relationships, for any label *s*, the membership function maps each element of the universe of discourse to a real number in the interval. Theoretical research on semantic space properties [33,34] has formulated the following requirements for the membership functions:

(a) Each label has a normal membership function ($\forall \;s\in S,\;\exists \;x\in U:\mu_{S}\left(x\right)=1$);
(b) The term set *S* is a Ruspini partition, ($\forall \;x\in U,\;\sum_{i=1}^{n}\mu_{s_{i}}\left(x\right)=1$), which ensures, for each unit from the universal set, the availability of at least one concept that describes this unit with a nonzero grade of membership. Additionally, it ensures the discriminability of concepts generating the semantic space and excludes the use of synonyms or semantically close terms. Consequently, each element of the universe can be described by no more than two labels;
(c) In addition, the term set *S* must be complete: each element of the universal set can be described within the scope of at least one label.

Internal features of context are defined in terms of experts’ knowledge and pragmatics and introduce a dynamic behavior in semantics as it changes when information increases [35], for example, when modifiers or hedges are used. These dynamics are modeled by the set of semantic rules that are used to assign every generated label a membership function in [0,1] × [0,1], taking into account the static constraints imposed by external features of context. There are two different levels:

- The elicitation of the primary linguistic terms. According to linguistic theory, “the meaning of an adjective must be such that the comparative forms can be understood as a semantic transformation of that meaning into the right binary relation” [36]. Then, the primary term set includes the comparative forms of the adjective (*Less Important*, *Equally Important*, *More Important*). An adjective is a degree adjective if it can occur in a predicative position (i.e., after copular verbs, such as be, seem, become), and it can be preceded by degree modifiers (i.e., very and fairly). A degree adjective is linear if it is possible to construct a linear ordering of all objects in the domain of application of the adjective independently of the context [37]. As a result, the adjective *Important* is classified as a degree non-linear adjective. Linear adjectives (e.g., *tall*) exhibit a particular property called graduality, which is linked to the vagueness of a term: there is a gradual transition between objects identified as definitely true and those that are completely false. Nonlinear adjectives also exhibit a second property, indeterminacy, as the possibility of associating a single lexical item with several related measure functions [38]. We understand the determinacy of a linguistic term as the property that provides the amount of information contained in it. Possibility theory focuses primarily on this intrinsic imprecision of natural languages, and it assumes that this imprecision is a question of possibility rather than probability. Dubois [26] gives, through a transformation of a probability distribution into a possibility distribution, a natural interpretation of the symmetric triangular membership function that points is appropriate for the analytical expression of the above-mentioned graduality;
- The elicitation of the terms is obtained when linguistic modifiers are applied to the primary ones. The lack of precision of a linguistic term is not only a question of specificity but also the entropy of the term [39], defined as a measure of its applicability to a concept. In this context, the entropy definition of Rojas [40] gives a value for this measure:

(3) $$E\left(\mu_{S}\right)=\frac{\left|\mu_{S}\cap \mu_{S}{}^{C}\right|}{\left|\mu_{S}\cup \mu_{S}{}^{C}\right|}$$

Then, if a linguistic hedge increases the precision of the meaning when it is applied, the modified term must have lower entropy. This property allows us to move from the meaning of the most general term describing a preference to the strongest one describing the absolute preference with a fuzzy singleton number. This is the main reason to avoid the use of a uniformly distributed set of uniform membership functions.

Linguistic hedges or modifiers form a subclass of adverbials that enable us to represent small variations of imprecise characterizations of given linguistic variables on the basis of some properties [41]:

(a) Each modifier slightly changes the meaning of the original term. When the modifiers are applied to a primary term set or linguistic trichotomy [42], the original essential meaning is preserved. This is the so-called semantic heredity [43];
(b) Linguistic modifiers have two main behaviors with regard to their effect on the qualifications they modulate [18]. They act as either reinforcement (e.g., much, strongly) or weakening (e.g., hardly, moderately). The latter is influenced by the notion of the ordering proximity in the domain where they operate [29];
(c) The sets of hedges are partially ordered sets or posets due to their inherent meaning. (e.g., hardly < moderately < much < strongly). This ordering relation can be interpreted in two ways [44,45]:
  ○ In the inclusive interpretation, the semantic entailment holds (e.g., strongly much);
  ○ In the non-inclusive interpretation, terms denote different but possibly overlapping categories.

It is common to apply one interpretation to all the hedges used to modulate the meaning of a primary term set. We think that this modus operandi is wrong because each modifier has an intrinsic meaning (and interpretation), and the duplicity is then due to prior knowledge about the problem to which these modifiers are used. This issue results in a simplification of the verbal expression’s formulation (e.g., the adverb *much* applied to the term *more* defines a subset *much more* ⊆ *more*; the non-inclusive interpretation results from the simplification of the linguistic expression “*more but not much more*” being the hedge inclusively interpreted, in the term *more*). The requirement of Ruspini partitions is the pragmatic implication derived from the former statement. Nevertheless, the meaning of each modifier must be initially elicited by considering its intrinsic behavior.

Reinforcement hedges are inherently inclusive with regard to the primary term that they modify (e.g., *very important* things are also *important* things). Novak [46] uses this idea: “What does it mean “very small”? The hedge very makes the meaning of small numbers more accurate. Very small numbers are small, but there are small numbers which are not very small”.

For the weakening hedges, we found that there is a conceptual conflict in the semantic entailment between weakening and reinforcement hedges. For instance, when inclusive interpretation is considered, it is accepted that *strongly* ⊆ *much moderately* ⊆ *hardly*. Although mathematically, there is evidence that this assessment implies that strongly is a subset of hardly, we uphold that, at the linguistic level, it is not acceptable to include the meaning of a reinforcement adverb in the meaning of a weakening one, e.g., something that is *strongly preferred* is not also *hardly preferred*; they belong to different psychological categories. Thinking of hedges as *moderately*, *slightly*, and *hardly*, one immediately observes that there is an inherent component of similarity with regard to the term they modify and with its complementary (e.g., the meaning of “*hardly different*” is very close to “*equal*”). This dual proximity confers the non-inclusive characteristic of weakening hedges with regard to the primary term and subordinates the corresponding elicitation to a previous estimation of their natural position in the universe of discourse.

## 3. Term Set Elicitation Procedure

### 3.1. Primary Term Set Elicitation: Semantics for Comparative Forms of the Adjective Important

The correct way to assign a membership function for each label of the primary term set requires starting from a referential term with a clear semantic meaning. For preferences, this term is the corresponding term that describes the Indifference between criteria or alternatives. In grammar *G*, this term is Equally Important. There are some properties about its membership function that we can establish “a priori”:

- It has a natural positioning. The ordered structure imposes that the membership function for the label Equally Important is symmetrically centered in the universe of discourse *U*;
- It must be a normal membership function. The maximum degree of indifference between any alternative ci and itself imposes a degree of membership for *u* = 0.5 (the core of the membership function);
- It must cover the universe of discourse with correspondence with a maximum level of indeterminacy (or minimum value of specificity). This property is closely related to completeness [47].

Zadeh [48] showed that a membership function of a fuzzy set could be used to encode a possibility distribution, and in Zadeh’s view, possibility distributions were meant to provide graded semantics to natural language statements. We use this working line to determine the shape to model the graduality of the referential term Equally Important.

There are two necessary conditions to apply this procedure:

- The associated probability distribution function must be uniform;
- The most representative value for the uniform probability distribution must be the mean value of the support interval.

For the first condition, the most natural probabilistic representation of incomplete knowledge (maximum indeterminacy scenario) when only the support is known is the uniform distribution of the universe of discourse; for the second one, if we think in the linguistic term, Equally Important, for which the semantics are being analyzed, it is evident that *u* = 0.5 plays this role. Consequently, the elicitation of the reference term Equally Important requires a symmetric triangular membership function. In a scenario of maximum indeterminacy, the support of this triangular membership function is the interval [0,1]. Because this line of argumentation is also valid for any support [*x_m−δ_*, *x_m+δ_*] of the possibility distribution function, the symmetric triangular shape of the referential term membership function remains with lower levels of indeterminacy. This assessment is a key point of our model: because the application of a hedge or modifier makes the meaning of a verbal expression more precise, the higher the number of hedges used by an expert, the higher the information content by the corresponding terms and the narrower the associated support of the membership function. In other words, the specificity of the referential term is not arbitrary because it is conditioned by the number and type of modifiers used by the experts; however, its shape is always triangular and symmetrical.

**Figure 1:**
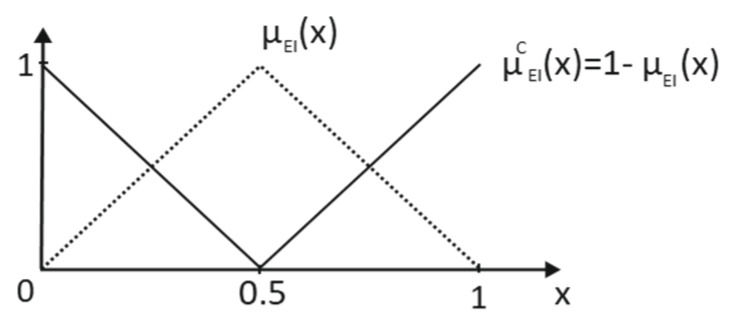
Referential term elicitation and its complementary (maximum indeterminacy scenario).

The primary terms More Important (*MI*) and Less Important (*LI*) are obtained from the complementary of Equally Important (*EI*), as it is shown in Figure 1 and Equation (4):

(4) $$\mu_{EI}^{C}\left(x\right)=1-\mu_{EI}\left(x\right)\;\;\Rightarrow \{\begin{array}{c} \mu_{LI}\left(x\right)=\{\begin{array}{c} \mu_{EI}^{C}\left(x\right),\;\;x\in \left[0,0.5\right]\\ 0\;elsewhere \end{array}\\ \mu_{MI}\left(x\right)=\{\begin{array}{c} \mu_{EI}^{C}\left(x\right),\;\;x\in \left[0.5,0\right]\\ 0\;elsewhere \end{array} \end{array}\;\;\;$$

### 3.2. Non-Primary Term Set Elicitation: Semantics for Linguistic Modifiers or Hedges

#### 3.2.1. Influence of Hedges in Primary Term Set Meaning

In the late 90s, two new models emerged to provide clear semantics to the hedge membership functions: the horizon shifting model introduced by Vilém Novak [29] and the fuzzy relational-based model of Martine DeCock and Etienne Kerre [49].

Novak’s horizon-shifting model is based on the concept of a horizon, which represents the border between finite and infinite numbers in Alternative Set Theory. In this frame, infinity is, in some sense, a synonym for non-transparency because the position of the horizon depends on the ability to verify the finiteness of a number. This concept is reinterpreted by Novak [42], who elicits modifiers by an approach function between the horizon and observer positions linked to the support and core of the corresponding membership functions. We use this procedure to assign the meaning of labels containing reinforcement hedges. In De Cock’s fuzzy relational calculus-based model, the semantics of modified linguistic expressions is obtained by taking into account the mutual relationships between the objects of the universe that are formally defined as fuzzy resemblance relations (images of fuzzy sets under fuzzy relations). We use this method to assign the meaning of labels containing weakening hedges.

The approach and resemblance functions can be implemented through a pseudo-metric d:U×U→ℜ where d(x,x)=0, d(x,y)=d(x,y) and d(x,y)+d(y,z)≥d(x,z) for all x,y,z∈U. Let (U,d) be pseudo-metric space and let ν, b and h be three elements of U called observer, border and horizon, respectively. The elements of the universe that are closer than the border *b* at a distance *d_min_* from the observer are clearly “visible” (they have a membership degree 1), and those that are behind the horizon *h*, at a distance *d_max_*, are not “visible” at all (they have a membership degree 0). If d(ν,x)≤d(ν,y), then the observer views clearer *x* than *y* (the membership degree of *x* is greater than the one that corresponds to *y*):

(5) $$\mu \left(x\right)=\{\begin{array}{ccc} 1 & if & d\left(\nu ,x\right)\leq d_{min}\\ decreasing & if & d_{min}<d\left(\nu ,x\right)\leq d_{max}\\ 0 & if & d\left(\nu ,x\right)>d_{max} \end{array}\;\;\;$$

For a linear transition with d(x,y)=|x−y|:

(6) $$\mu \left(x\right)=\{\begin{array}{c} 1\;\;\;\;\;\;\;\;\;if\;\left|\nu -x\right|\leq \left|\nu -b\right|\\ 1-\frac{\left|\nu -x\right|-\left|\nu -b\right|}{\left|\nu -h\right|-\left|\nu -b\right|}\;\;if\;\left|\nu -b\right|<\left|\nu -x\right|<\left|\nu -h\right|\\ 0\;\;\;\;\;\;\;\;\;if\;\left|\nu -x\right|\geq \left|\nu -h\right| \end{array}\;\;\;$$

Then, the elicitation of any linguistic label requires determining the position of the observer, the horizon and the border (in fuzzy classical terminology, they define the support and the core of the corresponding membership function).

Dealing with preferences, observer, and border position matches for the reference term Equally Important in the maximum indeterminacy scenario ($\nu_{EI}^{\left(0\right)}=0.5$,$b_{EI}^{\left(0\right)}=0.5$). Horizons position corresponds with the maximum degree of preference placed at the furthest distance ($h_{EI,L}^{\left(0\right)}=0$ and $h_{EI,R}^{\left(0\right)}=1$). At this level, Table 1 resumes the elicitation of the primary term set:

In the former situation, $d_{EI,min}^{\left(0\right)}=0$ and $d_{EI,max}^{\left(0\right)}=0.5$. When hedges are introduced, in the reference term, the observer sees the horizons closer, $d_{EI,max}^{\left(1\right)}$ decreases, and the entropy of this reference term is then lower. Because of the triangular shape that we have chosen as the best to elicit this term, the border does not change its position and $d_{EI,min}^{\left(1\right)}=0$.

**Figure 2:**
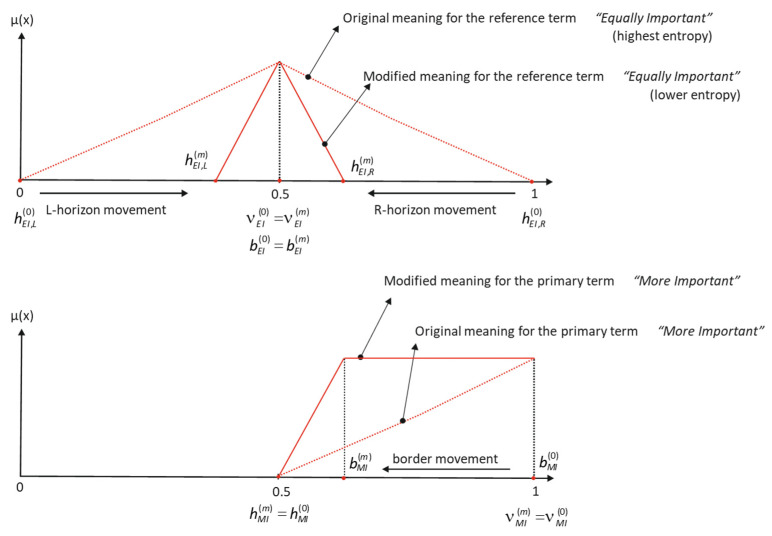
Influence of hedges in primary term set meaning.

As we can graphically see in Figure 2, the change in the horizon position in the reference term causes a movement in the border of the complementary terms *More/Less Important*.

The pragmatic interpretation leads us to state that higher linguistic precision allows a greater distance between the observer and border to clearly define what *More/Less Important* are (the term specificity increases and the corresponding entropies decrease).

In the proposed grammar, we only applied hedges to those terms indicating a positive or a negative degree of preference. In the rest of the paper, we describe the procedure when it is applied to terms indicating a positive preference; the results and the negative values are symmetrical about *x* = 0.5.

#### 3.2.2. Reinforcement Hedges Mainstreaming

This second step of the elicitation procedure requires the completion of the set of values to give a concrete meaning for the subset of linguistic labels obtained, excluding those that contain weakening hedges. In addition to their inclusive character, we consider that the graduality slope increases with the modifier’s strength to obtain a smooth transition between the meaning of the primary term More Important and the modified term indicating an absolute preference. We have chosen the following numerical sequences to determine the horizon and border positions of the modified preference terms:

(7) $$\begin{array}{c} b_{MI_{n}}^{\left(1\right)}=\nu_{EI}^{\left(1\right)}+2\cdot \frac{1-\nu_{EI}^{\left(1\right)}}{k\cdot \left(k+1\right)}\cdot \left(n\cdot k-\sum_{i=1}^{n}\left(i-1\right)\right)\\ h_{MI_{n}}^{\left(1\right)}=\nu_{EI}^{\left(1\right)}+2\cdot \frac{1-\nu_{EI}^{\left(1\right)}}{k\cdot \left(k+1\right)}\cdot \left(\left(n-1\right)\cdot \left(k+1\right)-\sum_{i=1}^{n}\left(i-1\right)\right)\;\;\; \end{array}$$

where:

- *n* = degree of strength;
  *MI*_1_: *More Important (MI)*; *MI*_2_: *Much More Important (MUMI)*;
  *MI*_3_: *Strongly More Important (SMI)*; *MI*_4_: *Extremely More Important (EMI)*.
- *k* = number of labels describing a positive preference, excluding those with weakening hedges.

**Figure 3:**
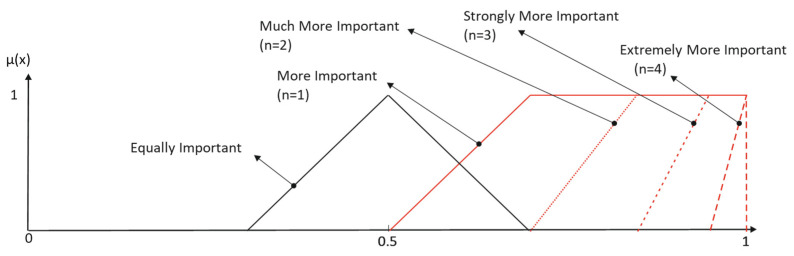
Inclusive elicitation with only reinforcement modifiers.

Figure 3 and Table 2 show the results obtained when the modifiers *Much*, *Strongly* and *Extremely* are applied to the primary term *More Important* when inclusive interpretation is considered.

**Figure 4:**
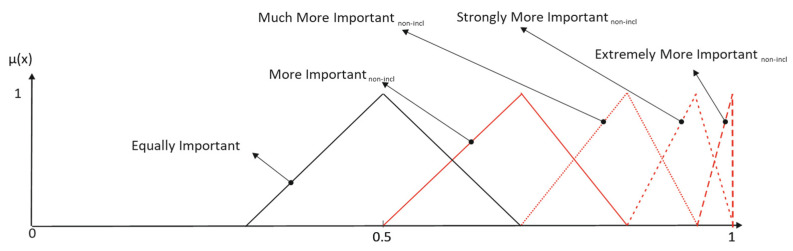
Non-inclusive elicitation with only reinforcement hedges.

Desirable semantic space properties require that each element of the universe be described by no more than two linguistic terms, and the membership functions must result in a Ruspini partition. Then, a non-inclusive set of membership functions is sought. Consequently, the labels with reinforcement hedges must be reinterpreted, as shown in the following example (Figure 4):

(8) $$\mathrm{More}\;{\mathrm{Important}}_{\text{non-incl}}=\mathrm{More}\;\mathrm{Important}\cap {\left(\mathrm{Much}\;\mathrm{More}\;\mathrm{Important}\right)}^{C}$$

#### 3.2.3. Weakening Hedges Mainstreaming

The weakening modifiers provide a new characterization that is less strong than the original one [50]. In preference modeling, weakening hedges, such as moderately and hardly, are used to describe situations where there are no significant differences between the compared alternatives, but it is assumed that this difference exists. Therefore, the meaning given to the modified terms, including weakening hedges, is associated with a similarity measure between the meanings of the primary term set labels. For this purpose, De Cock and Kerre [51] defined the notion of the resemblance relation to model the vague concept of approximate equality through its intuitive connection to the distance concept in those situations where it is hard to tell whether objects are approximately equal. They also propose this model to avoid the non-transitivity of the approximate equality (or Poincaré paradox [52]).

A fuzzy relation *E* on a pseudo-metric space (*U,d*) is called a resemblance function *E* if for all *x, y, z* and *u* in *U* [38]:

(9) $$\begin{array}{c} E\left(x,x\right)=1\\ d\left(x,y\right)\leq d\left(z,u\right)\;implies\;E\left(x,y\right)\geq E\left(z,u\right) \end{array}$$

With d(x,y)=|x−y|, a general definition for these relations is given by:

(10) E(x,y)=min[1,max(0,A−B·|x−y|)]

where *A* and *B* define the length of the kernel and support, respectively.

In De Cock’s representational scheme [49], the modified linguistic expressions have clear semantics by taking into account the mutual relationships between the objects of the universe. These resemblance relationships depend on the nature of the modifier. For weakening hedges, overlapping (which is linked with the T-intersection of fuzzy sets) between the resemblance function and the membership function of the original term is considered adequate. In our case, the application of these concepts requires first considering the hierarchical structure of the weakening hedges used in the proposed grammar (*Hardly*, *Weakly* and *Moderately*):

- For the first level, a weakened meaning of the label *More Important* must include the elements that belong to these labels but also resemble the meaning given to the label Equally Important. Then, the resemblance function considered places its core in *x* = 0.5 and has a support length $\left|b_{EI,L}-b_{EI,R}\right|$:

(11) $$E\left(x,0.5\right)=\{\begin{array}{c} \min\left[1,\max\left(0,\;1-\left|0.5-b_{EI,L}^{\left(1\right)}\right|\cdot \left|x-\nu_{EI}^{\left(1\right)}\right|\right)\right]\;;\;x\leq 0.5\\ \min\left[1,\max\left(0,\;1-\left|0.5-b_{EI,R}^{\left(1\right)}\right|\cdot \left|x-\nu_{EI}^{\left(1\right)}\right|\right)\right]\;;\;x>0.5 \end{array}$$

The results obtained by the overlapping of E(x,0.5) and the membership function for *More Important* give us the observer position for the *Weakly More Important* label. If the Gödel intersection is used as the overlapping operator:

(12) $$\nu_{WMI}^{\left(2,1\right)}=x\in U:\;\max\left\{\;\min\left[E\left(x,0.5\right),More\;{\mathrm{Important}}^{\left(1\right)}\left(x\right)\right]\right\}$$

Because normalized membership functions and Ruspini partitions are required, the elicitation of the *Weakly More Important* label implies that the horizons and borders of the primary term set change their position. The semantic subspace where they have to be elicited becomes narrower.

Borders and horizons of reinforcement edges should be recalculated as follows:

(13) $$\begin{array}{c} {b_{MI_{n}}^{\left(2,1\right)}=\nu_{WMI}^{\left(2,1\right)}+2\cdot \frac{1-\nu_{WMI}^{\left(2,1\right)}}{k\cdot \left(k+1\right)}\cdot \left(n\cdot k-\sum_{i=1}^{n}\left(i-1\right)\right)\;|}_{n}\\ h_{MI_{n}}^{\left(2,1\right)}=b_{WMI}^{\left(2,1\right)}+2\cdot \frac{1-\nu_{WMI}^{\left(2,1\right)}}{k\cdot \left(k+1\right)}\cdot {\left(\left(k+1\right)\cdot \left(n-1\right)-\sum_{i=1}^{n}\left(i-1\right)\right)|}_{n} \end{array}$$

**Figure 5:**
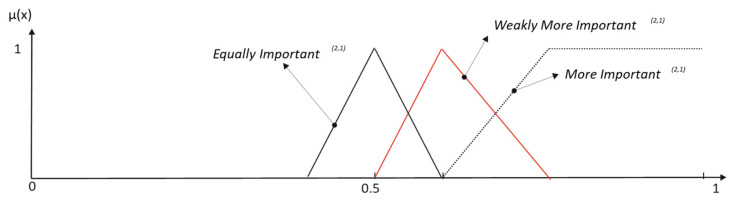
Elicitation of the first level of weakening.

Then, we have the following results (Table 3 and Figure 5):

- Once the label *Weakly More Important* is elicited, a second hierarchical level of weakening modifiers can be considered. In our grammar, these modifiers are *Hardly* and *Moderately*. To define their meaning, we apply the same procedure:
  ○ *Hardly More Important* must include the elements that belong to the general weakening label *Weakly More Important* but also resemble the meaning given to the label Equally Important. The resemblance function used is then:
    (14) $$E^{\prime }\left(x,0.5\right)=\{\begin{array}{c} \min\left[1,\max\left(0,\;1-\left|0.5-h_{EI,L}^{\left(2,1\right)}\right|\cdot \left|x-\nu_{EI}^{\left(2,1\right)}\right|\right)\right]\;;\;x\leq 0.5\\ \min\left[1,\max\left(0,\;1-\left|0.5-h_{EI,R}^{\left(2,1\right)}\right|\cdot \left|x-\nu_{EI}^{\left(2,1\right)}\right|\right)\right];\;x>0.5 \end{array}$$
  ○ *Moderately More Important* must include the elements that belong to the meaning given to the label *More Important* but also resemble the general weakening label *Weakly More Important*. The corresponding resemblance function is:
    (15) $$E^{\prime \prime }\left(x,\nu_{WMI}^{\left(2,1\right)}\right)=\{\begin{array}{c} \min\left[1,\max\left(0,\;1-\left|\nu_{WMI}^{\left(2,1\right)}-h_{WMI,L}^{\left(2,1\right)}\right|\cdot \left|x-\nu_{WMI}^{\left(2,1\right)}\right|\right)\right]\;;\;0.5\leq x\leq \nu_{WMI}^{\left(2,1\right)}\\ \min\left[1,\max\left(0,\;1-\left|\nu_{WMI}^{\left(2,1\right)}-h_{WMI,R}^{\left(2,1\right)}\right|\cdot \left|x-\nu_{WMI}^{\left(2,1\right)}\right|\right)\right];\;\nu_{WMI}^{\left(2,1\right)}<x\leq 1 \end{array}$$

With the same constraints, the observer positions for the new two labels are:

(16) $$\begin{array}{c} \nu_{HMI}^{\left(2,2\right)}=x\in U:\;\max\left\{\;\min\left[E^{\prime }\left(x,0.5\right),Weakly\;{\mathrm{Important}}^{\left(2,1\right)}\left(x\right)\right]\right\}\\ \nu_{MoMI}^{\left(2,2\right)}=x\in U:\;\max\left\{\;\min\left[E^{\prime \prime }\left(x,\nu_{WMI}^{\left(2,1\right)}\right),More\;{\mathrm{Important}}^{\left(2,1\right)}\left(x\right)\right]\right\} \end{array}$$

The new borders and horizons of reinforcement edges are obtained by:

(17) $$\begin{array}{c} {b_{MI_{n}}^{\left(2,2\right)}=\nu_{MoMI}^{\left(2,2\right)}+2\cdot \frac{1-\nu_{MoMI}^{\left(2,2\right)}}{k\cdot \left(k+1\right)}\cdot \left(n\cdot k-\sum_{i=1}^{n}\left(i-1\right)\right)\;|}_{n}\\ h_{MI_{n}}^{\left(2,2\right)}=b_{MoMI}^{\left(2,2\right)}+2\cdot \frac{1-\nu_{MoMI}^{\left(2,2\right)}}{k\cdot \left(k+1\right)}\cdot {\left(\left(k+1\right)\cdot \left(n-1\right)-\sum_{i=1}^{n}\left(i-1\right)\right)|}_{n} \end{array}$$

**Figure 6:**
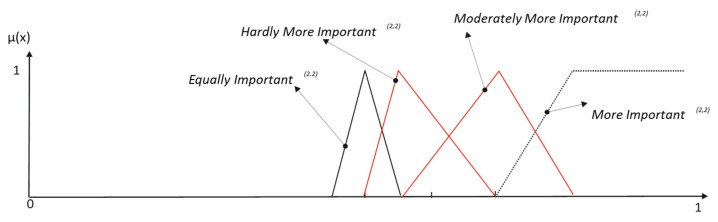
Elicitation of the second level of weakening.

The complete results of the elicitation are shown in Table 4 and Figure 6.

## 4. Discussion

If the linguistic approach is used for approximate reasoning, its fundamentals (the linguistic values or labels and their elicitation) must be supported by arguments that go beyond the simplicity or the extended use of certain types of mathematical functions. The required mathematical function assignment to a linguistic term should be based on linguistic semantics and pragmatics principles. For preference relationships and pair-wise comparisons, there are thousands of papers using triangular membership functions uniformly distributed in the universe of discourse with no apparent reason. This practice is not desirable when decisions are going to be made in real situations to solve real problems. Our aim was to obtain a set of rules to assign a membership function to each linguistic value with an at least psychologically reasonable linguistic explanation. Thus, applied linguistics basic principles and definitions should be taken into account when the fuzzy linguistic approach is applied.

**Figure 7:**
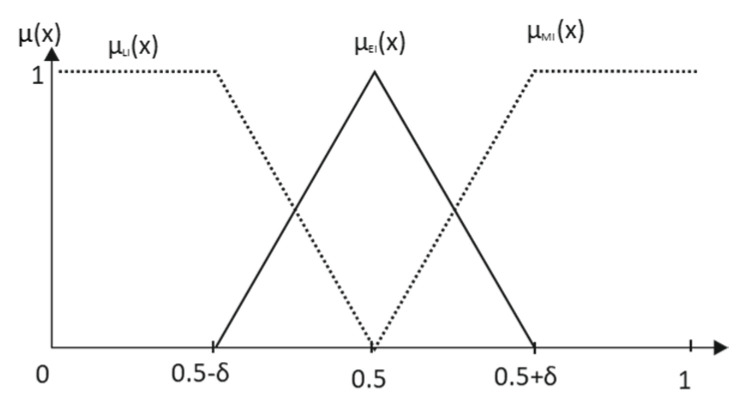
Elicitation of the primary term set.

In the frame of pairwise comparisons, the external features of linguistic context, which are independent of the expert group, include the semantic space usually considered and the desirable properties for the fuzzy partition derived from the assignment. In the semantic space [0,1] × [0,1], possibility theory allows eliciting the so-called reference term *Equally Important* with clear semantics [26,34] being the linear transition around 0.5 the only acceptable solution for graduality modeling for any support [0.5 − *δ*, 0.5 + *δ*] of the referential term (Figure 7).

The internal features of linguistic context, which are dependent on the amount of information introduced by the agents (experts), introduce a dynamic behavior in meanings and are also related to language complementarity. Hedges or modifiers are the most visible linguistic signs of the internal context.

**Figure 8:**
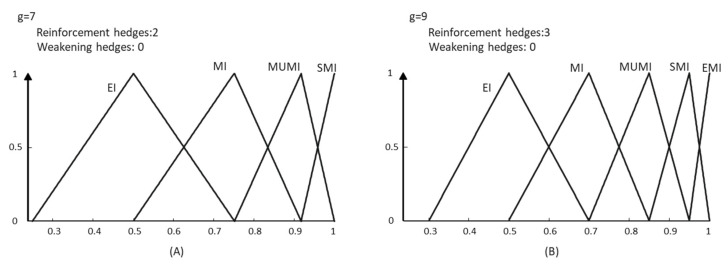
Change in fuzziness when the number of hedges increases. (**A**) Elicitation with 2 reinforcement hedges. (**B**) Elicitation with 3 reinforcement hedges.

Lakoff’s assessment “Hedges make meanings fuzzier or less fuzzy” [3] is the basis of almost every published work on the semantic interpretation of modifiers (for example, the method developed by DeCock and Kerre in their relational model [30]). Consequently, the studies identify the weakening effect with an expansive behavior and the reinforcement effect with a restrictive behavior. These models apply the modifiers to linear adjectives (e.g., old, tall, heavy) whose main source of fuzziness is vagueness (expressed by the graduality). However, non-linear adjectives (such as *Important*) have another property linked to the imprecision of terms when the relativity to circumstances of the evaluation is considered, the so-called semantic indeterminacy, defined by Kennedy [38] as the possibility of associating a single lexical item with several but related measure functions. Nevertheless, graduality is subordinated to indeterminacy. This interaction restricts the validity of Lakoff’s assessment: when we add a term that has to be elicited in the same semantic space as the primary terms, the resulting meanings for the whole label set become less fuzzy. In this frame, the entropy of the linguistic terms’ meaning must decrease when modifiers are applied, as it is shown in Figure 8.

In the current literature, granularity is the only parameter used to modify the meaning of a label. However, our method also differentiates the character of the modifiers. In our framework, the meanings for labels, such as Hardly More/Less, are psychologically conditioned by the proximity of Equally, and the corresponding rule to elicit their membership functions must include this circumstance, which is the reason we uphold that linguistic interpretation for weakening modifiers is non-inclusive. Fuzzy relational calculus is considered a reliable tool to give meaning to labels with weakening hedges.

Nevertheless, inherent linguistic interpretation for intensive modifiers is inclusive. Consequently, their membership function assignment followed a different direction. The key statement that is the basis of our model for reinforcement hedges considers that the higher the hedge strength is, the lower the specificity and the entropy of the modified term must be. This assessment is supported by the psycholinguistic studies of Hersh and Caramazza [45], contrary to what is proposed in the current literature. For example, DeCock [51] states that the results of the abovementioned work should be taken as a reference to consider that the slopes remain unchanged when modifiers are applied, but if Hersh and Caramazza’s empirical outcomes were normalized, the conclusions drawn indicate the opposite.

**Figure 9:**
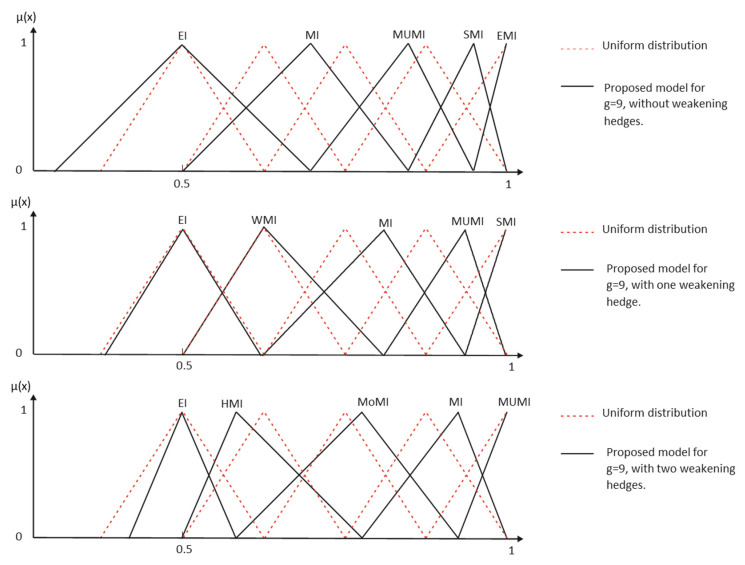
Results comparison with a uniform distribution of membership functions.

As a result, in our model, meanings are more dynamic, and the language complementarity problem has a better treatment. For example, for the proposed grammar, a typical term set granularity *g* = 9 could be completed in five different ways with their corresponding meanings, three of which are shown in Figure 9.

It is not unusual to find problems whose assessments are better modeled by means of linguistic term sets that are not uniformly distributed, as decision-making is based on pair-wise comparisons; that is the problem that we are facing. In these situations, it would be necessary to elicit preferences with different granularities, depending on the expert who assesses the problem.

When using two-tuple symbolic models, a previous definition of a linguistic hierarchy and a scale superposition algorithm is required to elicit the linguistic labels or terms, which results in a rigid solution where regardless of which linguistic term we link to which position *s_j_*, the final result is the same.

**Figure 10:**
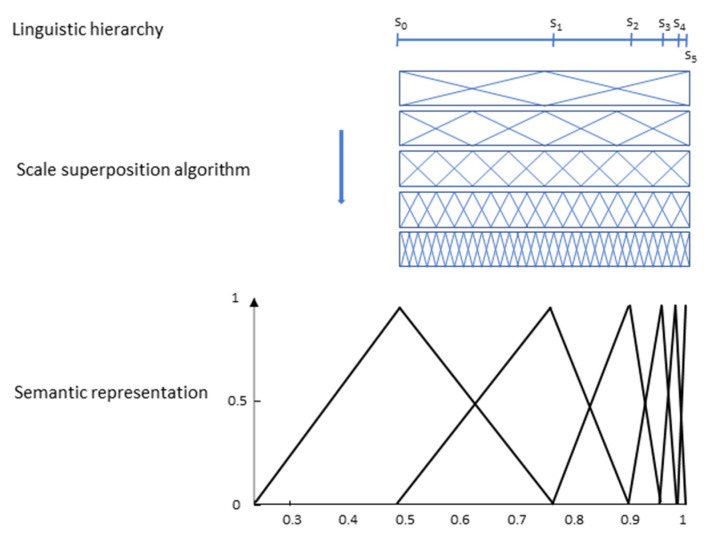
Example of semantic for a non-uniform distribution in two-tuple models.

This procedure seems to fit well working with low granularities, but acceptable linguistic interpretation becomes more difficult when g increases because functions tend to be concentrated, as we can see in Figure 10 for a term set with *g* = 11. This situation describes the problem of rigidity of this type of model, as outlined in the Introduction section.

**Figure 11:**
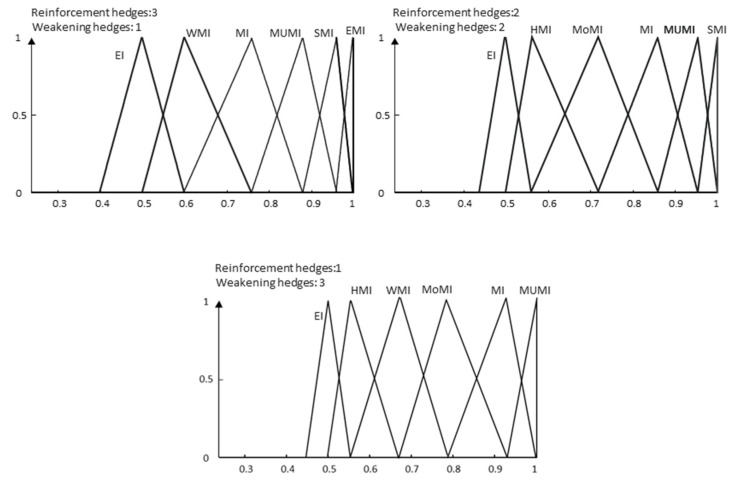
Semantics for three different scenarios.

In contrast, for the same granularity, our model allows the generation of several scenarios, depending on the hedges used by experts (Figure 11), making more flexible the interpretation of the results.

**Figure 12:**
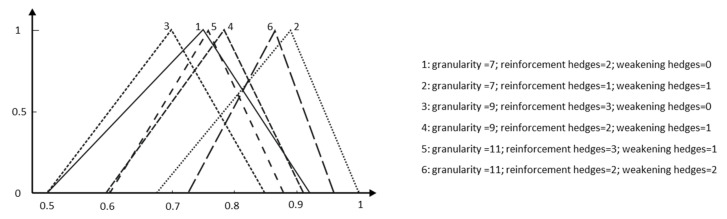
Semantics and their syntactic contexts for the term More Important.

Analogously to classical lexicographical tools, it could be useful to assemble the syntactic and semantic characteristics of the lexical units to find the best meaning for each context (Figure 12).

## 5. Conclusions

When designing a fuzzy linguistic preference model, there are two key points to consider: the elicitation of the membership functions assigned to each linguistic term and the method to analyze the consistency of the experts’ opinions through transitivity definitions. For the first objective, we uphold that the required mathematical function assignment to a linguistic term should be based on linguistic semantics and pragmatics principles.

In the frame of pairwise comparisons, the external features of the linguistic context were analyzed, and, as a result, linear transitions were adopted as the only option to model graduality and a triangular fuzzy number around 0.5 was assigned to elicit the referential term *Equally Important*. We start our contribution from these accepted statements.

With regard to internal features of linguistic context and according to linguistic complementarity, membership functions of the same concepts used by different people do not necessarily coincide. To address this principle, the most common parameter used to differentiate experts’ opinions is the granularity of the term set S. In our model, the weakening or reinforcement character of linguistic modifiers conditions the assignment process: supported by psycholinguistic studies, for reinforcement hedges, we consider that the higher the hedge strength is, the lower the specificity and the entropy of the modified term must be; in contrast, weakening modifiers provide a new characterization that is less strong than the original one and, consequently, we esteemed necessary to reflect the fact that there is an inherent component of similarity with regard to the term they modify and with its complementary. As a result, in our model, meanings are more dynamic, and the language complementarity problem has a better treatment.

## Figures and Tables

**Table 1:** Initial values of the model parameters for the primary term set.

Linguistic Term	Observer, Border and Horizon Position
Equally Important	$\nu_{\mathrm{EI}}^{\left(0\right)}=0.5$, $b_{\mathrm{EI}}^{\left(0\right)}=0.5$, $h_{\mathrm{EI},L}^{\left(0\right)}=0$, $h_{\mathrm{EI},R}^{\left(0\right)}=1$
More Important	$\nu_{\mathrm{MI}}^{\left(0\right)}=1$, $b_{\mathrm{MI}}^{\left(0\right)}=1$, $h_{\mathrm{MI}}^{\left(0\right)}=0.5$
Less Important	$\nu_{\mathrm{LI}}^{\left(0\right)}=0$, $b_{\mathrm{LI}}^{\left(0\right)}=1$, $h_{\mathrm{LI}}^{\left(0\right)}=0.5$

**Table 2:** Reinforcement hedges: observer, border and horizon positions.

Linguistic Term	Observer, Border and Horizon Position
More Important^(1)^	$\nu_{\mathrm{MI}}^{\left(1\right)}=1$, $b_{\mathrm{MI}}^{\left(1\right)}=b_{{\mathrm{MI}}_{1}}^{\left(1\right)}=0.7$, $h_{\mathrm{MI}}^{\left(1\right)}=h_{{\mathrm{MI}}_{1}}^{\left(1\right)}=0.5$
Much More Important^(1)^	$\nu_{\mathrm{MUMI}}^{\left(1\right)}=1$, $b_{\mathrm{MUMI}}^{\left(1\right)}=b_{{\mathrm{MI}}_{2}}^{\left(1\right)}=0.85$, $h_{\mathrm{MUMI}}^{\left(1\right)}=h_{{\mathrm{MI}}_{2}}^{\left(1\right)}=0.7$
Strongly More Important^(1)^	$\nu_{\mathrm{SMI}}^{\left(1\right)}=1$, $b_{\mathrm{SMI}}^{\left(1\right)}=b_{{\mathrm{MI}}_{3}}^{\left(1\right)}=0.95$, $h_{\mathrm{SMI}}^{\left(1\right)}=h_{{\mathrm{MI}}_{3}}^{\left(1\right)}=0.85$
Extremely More Important^(1)^	$\nu_{\mathrm{EMI}}^{\left(1\right)}=1$, $b_{\mathrm{EMI}}^{\left(1\right)}=b_{{\mathrm{MI}}_{4}}^{\left(1\right)}=1$, $h_{\mathrm{EMI}}^{\left(1\right)}=h_{{\mathrm{MI}}_{4}}^{\left(1\right)}=0.95$

**Table 3:** Elicitation of the first level of weakening.

Linguistic Term	Observer	Border	Horizons
Equally Important^(2,1)^	$\nu_{\mathrm{EI}}^{\left(2,1\right)}=\nu_{\mathrm{EI}}^{\left(1\right)}=0.5$	$b_{\mathrm{EI}}^{\left(2,2\right)}=\nu_{\mathrm{EI}}^{\left(2,2\right)}=0.5$	$h_{\mathrm{EI},R}^{\left(2,2\right)}=b_{\mathrm{WMI}}^{\left(2,2\right)}\;;\;\;h_{\mathrm{EI},L}^{\left(2,2\right)}=b_{\mathrm{WLI}}^{\left(2,2\right)}$
Weakly More Important^(2,1)^	$\nu_{\mathrm{WMI}}^{\left(2,1\right)}=0.6$	$b_{\mathrm{WMI}}^{\left(2,1\right)}=\nu_{\mathrm{WMI}}^{\left(2,1\right)}=0.6$	$h_{\mathrm{WMI},L}^{\left(2,1\right)}\;=b_{\mathrm{EI}}^{\left(2,1\right)}=0.5\;;\;h_{\mathrm{WMI},R}^{\left(2,1\right)}=\;b_{\mathrm{MI}}^{\left(2,1\right)}=0.76$
More Important^(2,1)^	$\nu_{\mathrm{MI}}^{\left(2,1\right)}=\nu_{{\mathrm{MI}}_{1}}^{\left(2,1\right)}=1$	$b_{\mathrm{MI}}^{\left(2,1\right)}=b_{{\mathrm{MI}}_{1}}^{\left(2,1\right)}=0.76$	$h_{\mathrm{MI}}^{\left(2,1\right)}=h_{{\mathrm{MI}}_{1}}^{\left(2,1\right)}=0.6$

**Table 4:** Elicitation of the second level of weakening.

Linguistic Term	Observer	Border	Horizons
Equally Important^(2,2)^	$\nu_{\mathrm{EI}}^{\left(2,2\right)}=\nu_{\mathrm{EI}}^{\left(1\right)}=0.5$	$b_{\mathrm{EI}}^{\left(2,2\right)}=\nu_{\mathrm{EI}}^{\left(2,2\right)}=0.5$	$h_{\mathrm{EI},R}^{\left(2,2\right)}=b_{\mathrm{HMI}}^{\left(2,2\right)}=0.56\;;\;\;h_{\mathrm{EI},L}^{\left(2,2\right)}=0.44$
Hardly More Important^(2,2)^	$\nu_{\mathrm{HMI}}^{\left(2,2\right)}=0.56$	$b_{\mathrm{HMI}}^{\left(2,2\right)}=\nu_{\mathrm{HMI}}^{\left(2,2\right)}=0.56$	$h_{\mathrm{HMI},L}^{\left(2,2\right)}=\;b_{\mathrm{EI}}^{\left(2,2\right)}=0.5\;;\;h_{\mathrm{HMI},R}^{\left(2,2\right)}=\;b_{\mathrm{MoMI}}^{\left(2,2\right)}=0.7$
Mod. More Important^(2,2)^	$\nu_{\mathrm{MoMI}}^{\left(2,2\right)}=0.7\;$	$b_{\mathrm{MoMI}}^{\left(2,2\right)}=\nu_{\mathrm{MoMI}}^{\left(2,2\right)}=0.7\;$	$h_{\mathrm{MoMI},L}^{\left(2,2\right)}=\;b_{\mathrm{EI}}^{\left(2,2\right)}=0.56\;;\;h_{\mathrm{MoMI},R}^{\left(2,2\right)}=\;b_{\mathrm{MI}}^{\left(2,2\right)}=0.85$
More Important^(2,2)^	$\nu_{\mathrm{MI}}^{\left(2,2\right)}=\nu_{{\mathrm{MI}}_{1}}^{\left(2,2\right)}=1$	$b_{{\mathrm{MI}}_{1}}^{\left(2,2\right)}=$ 0.85	$h_{\mathrm{MI}}^{\left(2,2\right)}=h_{{\mathrm{MI}}_{1}}^{\left(2,2\right)}=0.7$

## Data Availability

Data sharing not applicable.
